# Sumoylation in Physiology, Pathology and Therapy

**DOI:** 10.3390/cells11050814

**Published:** 2022-02-26

**Authors:** Umut Sahin, Hugues de Thé, Valérie Lallemand-Breitenbach

**Affiliations:** 1 Center for Life Sciences and Technologies, Department of Molecular Biology and Genetics, Bogazici University, Istanbul 34342, Turkey; 2 Cirb, Collège de France, PSL Research University, Inserm U1050, Cnrs UMR 7241, 11 Place Marcelin Berthelot, 75005 Paris, France; hugues.dethe@inserm.fr; 3 Inserm U944-Cnrs UMR 7212, Université de Paris, 1 Avenue Claude Vellefaux, 75010 Paris, France; 4 AP-HP, Service d’Hematologie, Hôpital St. Louis, 1 Avenue Claude Vellefaux, 75010 Paris, France

**Keywords:** small ubiquitin-like modifier, ubiquitin, post-translational modification, stress, cancer, neurodegeneration, infection

## Abstract

Sumoylation is an essential post-translational modification that has evolved to regulate intricate networks within emerging complexities of eukaryotic cells. Thousands of target substrates are modified by SUMO peptides, leading to changes in protein function, stability or localization, often by modulating interactions. At the cellular level, sumoylation functions as a key regulator of transcription, nuclear integrity, proliferation, senescence, lineage commitment and stemness. A growing number of prokaryotic and viral proteins are also emerging as prime sumoylation targets, highlighting the role of this modification during infection and in immune processes. Sumoylation also oversees epigenetic processes. Accordingly, at the physiological level, it acts as a crucial regulator of development. Yet, perhaps the most prominent function of sumoylation, from mammals to plants, is its role in orchestrating organismal responses to environmental stresses ranging from hypoxia to nutrient stress. Consequently, a growing list of pathological conditions, including cancer and neurodegeneration, have now been unambiguously associated with either aberrant sumoylation of specific proteins and/or dysregulated global cellular sumoylation. Therapeutic enforcement of sumoylation can also accomplish remarkable clinical responses in various diseases, notably acute promyelocytic leukemia (APL). In this review, we will discuss how this modification is emerging as a novel drug target, highlighting from the perspective of translational medicine, its potential and limitations.

## 1. Introduction

### 1.1. Sumoylation: Basic Mechanisms and Major Players

Researchers, over the past decades, have identified several ubiquitin-like post-translational modifiers, all of which can dynamically regulate protein function [1,2]. Among these, sumoylation entails covalent and reversible attachment of one or more of the four (among five) human SUMO (small ubiquitin-like modifier) paralogs onto target proteins [3]. The discovery of SUMO dates back to 1995 when researchers first identified SMT3, the gene encoding the sole SUMO protein in *S. cerevisiae*, based on its requirement for centromeric functions and cell division [4]. This was rapidly followed by the discovery of mammalian SUMO (GMP1, SUMO1, Sentrin) by multiple groups and its identification as a post-translational modifier [5,6,7,8,9]. Today, we know that SUMOs exist in all eukaryotes, display multifunctional characteristics and orchestrate vital cellular activities both in normal physiological settings and under stress conditions. 

### 1.2. SUMO Proteins: Similarities with and Differences from Ubiquitin

**Figure 1:**
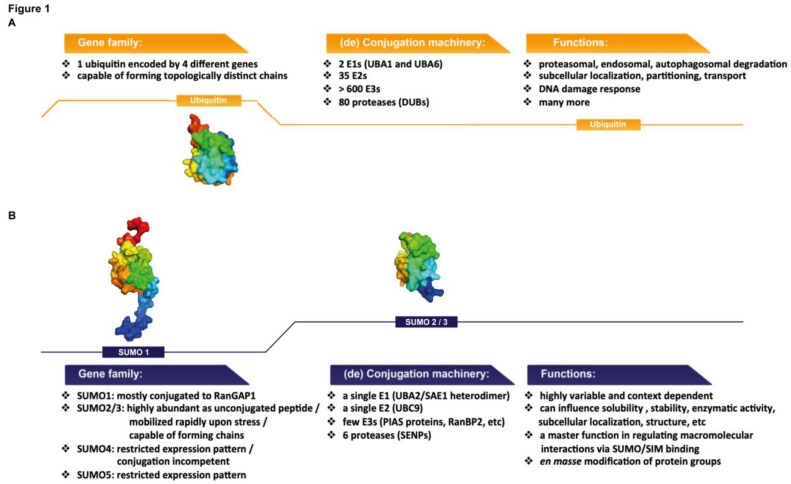
Similarities and disparities between ubiquitin (**A**) and SUMO (**B**), highlighted from a standpoint of structure, functional capabilities and the enzymatic machinery involved. Although SUMO1 shares an 18% amino-acid sequence identity with ubiquitin, the three-dimensional structures of SUMO1, SUMO2/3 and ubiquitin are remarkably similar, all featuring the central ubiquitin core that folds into a “ββαββαβ” pattern. The structure of SUMO1 is unique in that a flexible N-terminal extension protrudes from this central core (highlighted in dark blue). Images were generated by PyMOL (SUMO1 PDB: 2n1v, SUMO2/3 PDB: 2n1w, ubiquitin PDB: 1ubq).

The SUMO proteins are structurally related to ubiquitin, with SUMO1 displaying 18% amino acid sequence identity to the latter [10,11,12]. The core of SUMO1 is composed of a highly-noticeable, classical ββαββαβ “ubiquitin” fold. Both mature ubiquitin and SUMO proteins feature a C-terminal diglycine motif (-GG), which is engaged in a covalent isopeptide bond with the target lysine of protein substrates [1]. As for ubiquitin, the attachment can promote changes in substrate stability and be reversed by specific proteases. Despite the similarities between SUMO and ubiquitin (Figure 1A,B), a number of important differences exist, giving rise to some of the unique and fascinating biochemical and cellular functions attributed to SUMO. Critically, the surface charge distribution of SUMO is highly different from that of ubiquitin [13]. This unique electrostatic feature contributes to the exceptionally high solubility of the SUMO peptides and also facilitates binding interactions with a remarkably wide range of distinct proteins, in particular, those containing SUMO-interacting motifs (SIMs) [3]. In addition, a disordered N-terminal extension protrudes from the central “ubiquitin fold” of SUMO1, setting this peptide apart from ubiquitin and other ubiquitin-like modifiers [1,13]. Out of the five SUMO paralogs found in the human genome, SUMOs 1, 2 and 3 display ubiquitous expression patterns and have been extensively studied. SUMOs 2 and 3 share 97% sequence similarity and are together referred to as SUMO2/3. The latter shares 50% similarity with SUMO1 [3,13]. The functions of SUMOs 4 and 5 remain largely obscure. SUMO4, the expression of which is restricted to a few tissues including the kidney, spleen and the placenta, is likely not conjugation-competent as it contains a C-terminal proline residue (Pro 90) that precludes its protease-mediated maturation [14]. The expression of SUMO5 is limited to the testes and leukocytes and it may play a role in PML NB formation, though further studies are needed to establish this [15].

Although the enzymatic reactions are similar, another distinction with ubiquitin is the limited repertoire of sumoylation enzymes. The attachment of SUMO to proteins is implemented by a unique set of E1 and E2 enzymes, and in some cases, aided by a limited number of E3 ligases that may be instrumental in substrate recognition [3,16]. The classical sumoylation consensus motif is ψKxD/E (ψ denotes a large hydrophobic residue and x may be any residue), where UBC9, the E2 SUMO-conjugating enzyme, can readily dock [17,18]. Similar to SUMO, UBC9 itself features a unique surface charge distribution, displaying a strong electrostatic dipole [19,20,21,22,23]. The broad flexibility of UBC9 in target recognition is partially attributed to this quality, which consequently permits interactions with a surprisingly high variety of proteins. Indeed, extended, inverted or even post-translationally modified non-canonical sumoylation motifs have been identified on a wide range of substrates [16,24]. For instance, phosphorylation-dependent sumoylation motifs (PDSMs) contain a proline-directed phosphorylation site separated by two amino acids from a classical sumoylation consensus sequence, and are found in numerous transcriptional regulators [25,26]. Phosphorylation in this context increases the negative charge downstream of the SUMO-modified lysine residue, facilitating UBC9 interaction with the target substrate. Aside from the modified target protein, UBC9 also interacts with the E1 SUMO-activating enzyme (SAE1/UBA2 heterodimer in humans), which initially links a mature, conjugation-competent SUMO on UBC9 in an ATP-dependent manner [1,27,28,29]. Finally, contrary to the ubiquitination system in which a broad repertoire of E3 ligases are commissioned to assure specificity and enhance catalytic efficiency, only a handful of SUMO E3 ligases have been identified to date [3].

### 1.3. SUMO Proteases: The Dynamic Nature of SUMO Modification

Incidentally, the specificity of SUMOs’ detachment is also determined by a curiously limited number of enzymes, which are collectively referred to as SUMO proteases or SENPs [30,31]. SENPs are related to ubiquitin-like specific proteases (ULPs) and efficiently catalyze “desumoylation” of most substrates shortly after an initial burst of sumoylation. As such, sumoylation is one of the most dynamic protein modifications. In humans, the SENP family consists of six proteases (SENP1, 2, 3, 5, 6 and 7). Some of these display SUMO-paralog specificity for selective disengagement of SUMO1 versus SUMO2/3 from the substrates. Other SENPs may be confined to specific cellular compartments where they promiscuously detach any SUMO paralog from the substrates encountered [3].

This highly dynamic nature of sumoylation, along with its ability to target a large number of substrates by rapidly mobilizing a limited yet highly potent set of enzymes, renders it one of the most effective stress responders in the cell. We will discuss the relationship between SUMOs and cellular responses to environmental and pathological stressors in the following section. Most recently, large-scale comprehensive proteomic studies uncovered up to 14,000 endogenous sumoylation sites in human cells, revealing that thousands of structurally and functionally distinct proteins are indeed sumoylated [32]. Nonetheless, apart from a handful of proteins (i.e., RanGAP1) that absorb a large quantity of the otherwise limited supply of the unconjugated SUMO1 peptide, only a small fraction of most substrate proteins is modified by SUMO, especially in unstressed cells. Thus, sumoylation of most substrates is extremely challenging to detect. This observation is both intriguing and bewildering in that a tiny SUMO-modified fraction of a protein can accomplish a mass downstream effect [3,33,34,35]. For instance, for most transcription factors, sumoylation fulfills the function of an on-off switch of activity, where practically absolute transcriptional attenuation is driven by a tiny sumoylated fraction. This seemingly maximal effect accomplished by a small number of modified proteins may be rationalized by a number of mutually unexclusive models. One explanation is that an initial burst of sumoylation on a wide variety of targets facilitates the implementation of additional PTMs (i.e., acetylation or ubiquitination), leading to persistent effects on the entire target pool. In this scenario, SUMO moieties serve as a platform that recruits various types of enzymatic machinery, driving other modifications that remain on the substrate even after the SUMOs are cleaved off. For other substrates, the first wave of sumoylation may drag the target to a specific subcellular compartment or lock it into a protein complex, where the target remains even after the SUMOs are disengaged.

### 1.4. Protein Group Sumoylation Concept

A recently recognized feature of sumoylation is its ability to target entire protein groups within a complex in a highly selective and efficacious manner [3,36,37]. In these complexes, many functionally related proteins operate in a coordinated manner to accomplish a common goal. A topologically connected sumoylation enzyme modifies the whole group, where multiple SUMO moieties may serve as a glue, aided by non-covalent interactions with SUMO-interacting motifs (SIMs, see below). This way, although only a small fraction of the total pool is modified for each individual member, SUMO functionally controls the whole complex by achieving and sustaining its assembly. The protein group sumoylation concept is epitomized by the machinery that repairs DNA double-strand breaks in *S. cerevisiae* [38,39]. A DNA-bound SUMO E3 ligase, SIZ2, induces en masse sumoylation of the homologous recombination complex following genotoxic assault. Here, ablation of the sumoylation sites on specific members only mildly affects the damage response, whereas disabling the sumoylation cascade results in extreme vulnerability to DNA damaging stress. Importantly, several other subcellular sites and settings can serve as sumoylation hotspots where collective modification of functionally and topologically connected protein groups occurs en masse. These sites include several membranes-less organelles, such as stress granules and nuclear bodies (in particular, PML nuclear bodies, which will be further discussed in the stress section), the nucleolus and the telomeric regions [3,37]. Reciprocally, sumoylation may promote co-segregation of functionally unrelated proteins from the nucleoplasm to form topologically distinct droplets, a process unraveled by in vitro liquid-liquid phase separation experiments [40,41].

### 1.5. SUMO Chain Formation and the Role of SUMO-Interaction Motifs

Early evidence suggested that SUMO2/3 had a unique capacity for chain formation and could play distinct physiological roles from those of SUMO1. In fact, Lys11 of SUMO2/3 resides in a classical sumoylation consensus motif, meaning that this paralog itself is prone to its own sumoylation, forming SUMO2/3 chains on target substrates [42]. Mapping of the endogenous sumoylation sites in human cells was expedited by the advent of novel proteomic techniques, which revealed that aside from Lys11, other residues on SUMO2/3 including Lys7, Lys21 and Lys33 can also serve as internal SUMO attachment sites [43]. This means that SUMO2/3 chains can also engage in topologically distinct branching patterns, as observed with ubiquitin [44]. SUMO1 lacks an internal sumoylation consensus motif and was thought for many years to modify substrates solely via mono- or multi-modification (on multiple lysines), or to take part in poly-SUMO2/3 chains as a terminator cork. However, recent proteomic studies found that under stress conditions, multiple lysines on SUMO1, including Lys7 could be conjugated with SUMO2/3, although the role of these chains is still elusive [32,45].

The formation of poly-SUMO chains on target substrates begets the emergence of multiple weak interactions with various binding partners that often carry several SIMs [46]. SIMs have been found in a large number of proteins, most of which themselves are sumoylated. These SUMO-binding sequences can serve to dramatically modulate and enhance the interactor repertoire of sumoylated proteins, in turn, playing critical roles in cell signaling events both during development and under stress conditions. For example, the transcriptional repression of neural-specific genes in non-neuronal cells is achieved by the CoREST1/LSD1/histone deacetylase (HDAC) co-repressor complex, which is recruited to target promoter regions via a SIM sequence on CoREST1 that interacts with sumoylated chromatin-associated factors [47]. SIMs can also regulate paralog-specific SUMO conjugation to target substrates where a SIM-bearing protein might selectively recruit UBC9 carrying a particular SUMO isoform, as observed for substrates such as the ubiquitin-specific protease USP25, which is selectively modified by SUMO2/3 [48]. In addition, as mentioned above, SIM/SUMO interactions participate in the assembly and maintenance of protein complexes, including various DNA damage repair systems that form after genotoxic assault, and also of phase-separated membrane-less organelles, such as PML nuclear bodies. SIMs can play additional key roles in protein stability. Indeed, STUbLs (SUMO-targeted ubiquitin ligases) are a remarkable class of SIM-containing proteins [49]. They represent promiscuous ubiquitin ligases that make use of their multiple SIMs to recognize and ubiquitylate a wide variety of sumoylated targets, thereby inducing their degradation. In *S. cerevisiae*, the heterodimeric STUbL complex SLX5/SLX8 colocalizes to damage-induced DNA foci, such as double-strand breaks, along with many other sumoylated DNA repair enzymes [49,50]. RNF4 is a well-studied mammalian STUbL, which is also recruited to DNA damage foci following genotoxic stress [51]. Both yeast and human STUbLs are essential for cells to survive through genotoxic assault and are thought to expedite the clearance of the repair machinery components from these sites by inducing their degradative poly-ubiquitination. Poly-sumoylated PML (promyelocytic leukemia) protein was the first identified target substrate of RNF4 [52,53]. Today, we know that hundreds of such RNF4 substrates exist in mammalian cells [54].

To sum up, the SUMO system targets a large number of functionally and topologically connected proteins by employing a remarkably small number of enzymes, often in response to various cues. In addition, considering its capacity to impose a multitude of biochemical effects on its substrates and to drastically alter their interactor landscape, sumoylation appears a highly effective stress responder.

## 2. SUMO in Stress

How cells respond to stress has major implications for human physiology and pathology. Cells are frequently exposed to various kinds of exogenous and endogenous pressure, ranging from oncogenic assault to infections by pathogens, or from oxidative damage to nutrient depletion. Under stress, most cells mount an initial defensive response, which may result in the decoration of proteins with various post-translational modifications, institution of the unfolded protein response that involves chaperons among others, changes in gene expression, activation of the DNA damage repair systems, etc. If the extent of stress is prolonged, cells then mount long-term responses that may involve more profound epigenetic changes, metabolic shifts or activation of pathways leading to senescence or apoptosis [55,56]. Critically, most of these cellular systems and pathways are finely regulated by sumoylation.

PML nuclear bodies (NBs) are particularly interesting due their capacity to concentrate components of the sumoylation machinery, along with other enzymes that catalyze a variety of critical PTMs, as well as hundreds of other client proteins [57,58,59]. These peculiar structures form at the time of oligomerization of the scaffold PML protein, in particular, under stress conditions. Being a cysteine-rich protein, PML’s oligomerization is exquisitely sensitive to oxidation, highlighting the central physiological role of these membrane-less organelles in the coordination of cellular stress responses, particularly redox stress [60]. Following ROS-sensitive nucleation of the PML scaffold, multimeric PML then recruits UBC9, which rapidly catalyzes PML sumoylation [61]. Sumoylated PML is now capable of recruiting a broad range of client proteins with various key cellular functions, most of which carry one or more SIMs [59]. However, recent evidence indicates that PML NBs may also trap misfolded proteins that expose hydrophobic patches on their solvent accessible surface, or natively disordered ones [62]. As a biochemical consequence of client recruitment and local concentration at the PML core, catalysis of sumoylation occurs en masse, consequently igniting downstream PML-regulated action, such as survival via destruction of misfolded proteins, tumor suppression, senescence activation or transcriptional control [57,58,59]. This theory is best exemplified by p53 signaling: PML NBs recruit p53, along with many of its regulatory enzymes such as MDM2, CBP, HIPK2 and MOZ, forming a functionally and topologically connected protein group in situ [57]. Simultaneous sumoylation of these PML NB-concentrated proteins may enhance further intra-molecular engagements via SUMO/SIM interactions, to eventually accomplish full p53 activation through other relevant modifications [63]. Consequently, both PML and SUMO can act as key upstream regulators of p53, particularly in stress settings, and this PML/p53 axis is emerging as a critical mediator of cellular responses to oxidation, hypoxia, DNA damage and nutrient stress, as well as of senescence and tumor suppression during oncogenic assault [64,65]. Consistently, in mouse embryos, PML absence can promote survival at a time of exposure to the glutathione-synthesis inhibitor BSO, which normally induces loss-of-viability via apoptosis [64].

Aside from regulating multiple other p53 modifications, SUMOs may also act more directly as key transcriptional regulators, especially under oxidative or oncogenic stress. Indeed, the first link between sumoylation and transcription was established with the discovery that modification by SUMO1 enhanced p53 transcriptional activity [66,67]. SUMO1 conjugation of p53 may be facilitated by the critical p53 regulator ARF, in situ in PML NBs, resulting in the stabilization and activation of this important transcription factor, particularly during oncogenic assault, ultimately driving p53-dependent premature senescence [63]. While sumoylation induces the transcriptional activity of p53, for other transcription factors, it plays an antagonistic role. For example, modification of c-JUN by SUMO1 suppresses its transcriptional activity [68]. Furthermore, in other cases, sumoylation may indirectly activate some transcription factors that are crucial for mediating stress responses. HIF1 (hypoxia-inducible factor 1) is a chief transcription factor that mediates cellular responses to low oxygen levels, for example, during ischemia or tumor growth [69]. In normoxic conditions (when cells receive sufficient oxygen), the alpha subunit of HIF1 (HIF1α) is constitutively ubiquitinated by the VHL (von Hippel-Lindau) complex, a ubiquitin-E3 ligase, precipitating its proteasomal degradation [69]. Hypoxic conditions trigger VHL sumoylation by SUMO1, causing its inactivation [70]. This causes rapid stabilization of HIF1α, inducing the transcription of HIF1 target genes that counteract the lack of oxygen by initiating angiogenesis or enhancing the expression of glycolytic enzymes, among others.

The involvement of sumoylation in senescence or cell death pathways is not restricted to the regulation of p53 activity. In retrospect, the SUMO peptides were initially co-discovered as interaction partners of FAS and TNFR (tumor necrosis factor receptor), two key mediators of extrinsic apoptotic cues [6]. In addition, in the early days of the field, the modification of IκBα by SUMO1 was found to stabilize this protein complex by competing with its ubiquitylation on the same lysine residue, Lys21 [71]. IκBα functions as an inhibitor of NFκB. The latter is a transcription factor complex that induces apoptosis in response to various stimuli, such as TNF signaling, particularly under overwhelming stress conditions. Stress-induced sumoylation and stabilization of IκBα thus have a strong inhibitory effect on NFκB’s pro-apoptotic functions, allowing cells to remain alive and survive through distress until the initial assault is resolved.

Similar to oxidation, heat shock also inflicts considerable damage on cells, particularly harming proteins. Global SUMO conjugation swiftly responds to heat shock. Resting cells maintain only a limited amount of unconjugated SUMO1, with most of this peptide already quenched by proteins such as RanGAP1. On the other hand, in cell lines, free SUMO2/3 is abundantly expressed and rapidly mobilized following proteotoxic and hyperthermic stress to modify a wide range of target proteins [72,73,74]. Accordingly, both SUMO2 and SUMO3 were found to be essential for cells to survive through heat-induced damage [75]. Novel state-of-the-art proteomic techniques recently allowed researchers to detect the SUMO2/3 conjugation sites on endogenous proteins in cells exposed to hyperthermic stress. Under these conditions, SUMO2/3 was shown to form polymeric chains, within minutes, on thousands of proteins, particularly those that participate in cellular proteostasis, folding or degradation, revealing sumoylation as an immediate response to heat shock [75,76,77]. Globally increased sumoylation levels, in conjunction with the activation of chaperons and heat-shock proteins, can counteract the detrimental effects of stress-induced misfolding and be considered as a protective mechanism against protein aggregation. In addition, when polymeric SUMO2/3 chains form on misfolded proteins, the latter now become visible to STUbLs, such as RNF4, which can ultimately precipitate their destruction. Indeed, RNF4 silencing or proteasome inhibition can both result in the accumulation of cellular SUMO2/3 conjugates, implying that the latter are routinely destined for basal RNF4-induced ubiquitylation and proteasomal targeting [52,78,79]. Recent findings have also linked STUbL to the elimination of stress granules in the cytoplasm [40]. These membrane-less organelles are formed by co-accumulation of mRNAs and RNA-binding proteins under proteotoxic stress, such as heat shock or oxidation. Recent evidence indicates that multiple SUMO/SIM interactions may facilitate the assembly and maintenance of stress granules [40].

During genotoxic stress, as we briefly mentioned above, RNF4 functions as a critical component of the DNA damage repair machinery [51]. In addition, sumoylation itself has long been associated with chromosomal integrity and DNA repair [80]. As also stated earlier, DNA damage foci are hotspots for high sumoylation activity, and many distinct DNA repair systems, such as homologous recombination or nucleotide excision mechanism, all involve en masse sumoylation of individual components shortly after the damage is inflicted, followed by ubiquitylation-driven clearance from the repaired loci [3].

Bacterial and viral infections inflict substantial stress on cells and organisms. In this context, SUMOs have recently emerged as crucial components of innate immunity, not only regulating but also mediating host responses against infection [81,82,83,84,85]. Of note, SUMO peptides restrict pathogen replication. For instance, inhibition of cellular sumoylation can favor the replication of bacteria such as *Listeria monocytogenes*, or of viruses, including the human immunodeficiency virus (HIV) and the herpes simplex virus type 1 (HSV-1) [86,87,88]. On a molecular level, SUMO attachment can inhibit viral or bacterial proteins, which are normally needed for replication or latency control. On a cellular level, sumoylation regulates major immune-related signaling pathways, for example, those that are relayed by Toll-like receptors or interferon (IFN) receptors [87]. For example, many interferon regulatory factors (IRF) are subject to positive or negative regulation by sumoylation [81,84]. Interestingly, IFN signaling induces upregulation of both PML and SUMO protein levels, leading to enhanced sumoylation of many IFN-stimulated genes (ISGs) en masse in PML NBs [59,87]. Accordingly, silencing of PML or SUMO expression impairs interferon-mediated anti-viral effects, highlighting the importance of this PTM in first-line innate immune defenses [87]. On a physiological level, sumoylation also regulates T-cell expansion and activity, along with inflammatory responses [84]. Remarkably, during evolution, many pathogens have developed systems that can either disarm the host sumoylation cascade by attacking its critical enzymes, or induce proteolytic degradation of the sumoylated host proteins, thereby diminishing cellular sumoylation and impairing host immune responses [83,89]. In this respect, the HSV-1 protein ICP0 is paramount for viral targeting of host sumoylation. ICP0 is a STUbL, which can initiate proteasomal destruction of various sumoylated host proteins, particularly those with anti-viral functions, such as PML [88,90,91,92]. Similarly, a bacterium-encoded virulence factor, listeriolysin O (LLO), allows efficient replication of *Listeria monocytogenes* during infection by targeting the host UBC9 SUMO E2 enzyme [86]. Overall, sumoylation has now become a major theme in host-pathogen interactions and emerged as a focal point for various immune- and stress-related pathways that are activated during infections.

Finally, there is mounting evidence in support of a significant role for sumoylation in plants by providing resilience to stress conditions such as salinity, drought, nitrogen depletion, oxidation and infections [93,94,95]. Recent proteomic studies in Arabidopsis demonstrated that the plant SUMO system also modifies a large number of both nuclear proteins (including transcription factors, chromatin modifiers and DNA repair enzymes) and cytoplasmic ones, particularly those that function in protein synthesis, folding and degradation [94,95,96]. Exposing plant cells to various stress conditions invariably induces an upregulation in global sumoylation [97,98]. Furthermore, in-depth quantitative proteomic studies show that when plant cells encounter stress, the abundance of existing SUMO conjugates increases, rather than de novo modification of new substrates [99]. Intriguingly, these and many other studies suggest that manipulation of plant sumoylation by overexpressing the SUMO-conjugating enzymes or by silencing SUMO-specific proteases can enhance crop yield and provide protection against environmental stress. “SUMO engineering” is a rather new concept in agronomy and more research will be needed to fully appreciate its potential for enhanced stress tolerance in crops.

## 3. SUMO in Development and Physiology

Several recent studies have revealed a major role for SUMOs in epigenetic regulation and implementation of heritable changes in the chromatin, hence governing the cell identity, pluripotency, stemness and development [3]. The first link between sumoylation and epigenetics was established with the discovery that the N-terminal tail of histone H4 was post-translationally modified by SUMO [100]. Here, histone sumoylation resulted in transcriptional repression by facilitating the recruitment of histone deacetylases (HDACs) and the heterochromatin protein (HP1) to the modified locus. Over the years, growing evidence suggested that active sumoylation on the chromatin is associated with long-term repression of gene expression. Within this framework, sumoylation of the HP1 protein itself, which is mediated by Suv39h1 (a peculiar methyltransferase with its own SUMO E3 ligase activity), facilitates its association with the pericentric chromatin, a highly silenced region with extremely low transcriptional activity [101,102]. Similarly, DAXX functions as a chaperon for the H3.3 histone variant, which is tightly associated with the pericentric heterochromatin, and is known to be sumoylated [103,104]. The repressive KAP1/SETDB1 complex, which may involve HP1 protein, is also associated with the constitutive heterochromatin in other genomic regions, such as telomeres and transposable elements [105,106,107,108]. In line with the above observations, KAP1 multi-sumoylation seems to be critical for mediating interactions with its partners and its repressive activity [109,110,111,112]. Initially identified in Drosophila as repressors of the developmentally crucial homeotic genes, Polycomb group (PcG) proteins constitute an important class of epigenetic marks that are tightly linked with the silenced chromatin states in mammals. To mediate transcriptional repression, PcG proteins bind to each other and form large multimeric complexes on DNA that also recruit various transcription factors and co-repressors. Interestingly, the human Pc2 protein, a member of the PcG family, displays SUMO E3 ligase activity on various proteins, including the transcriptional co-repressor CtBP, suggesting that sumoylation may mediate PcG-driven chromatin silencing [113]. Senescent cells, where the expression of pro-proliferation genes is permanently silenced, also display abundant levels of sumoylation, which is required for the maintenance of the repressive H3K9Me3 histone mark on the chromatin [114]. More broadly, chromatin immunoprecipitation analyses of SUMO revealed sumoylation as a mechanism that sustains various chromatin states. Depending on the cell context, en masse sumoylation maintains active or inactive chromatin marks by tethering members of chromatin-remodeling complexes, as exemplified in pluripotent mouse embryonic stem cells (mESCs) or somatic induced pluripotent stem cells (iPSCs) [115]. Indeed, chromatin sumoylation is particularly involved in the maintenance of the identity of pluripotent stem cells. In addition, compared to differentiated cells, proteome sumoylation is also markedly enhanced in pluripotent cells and several principal transcription factors known to induce and maintain pluripotency, such as SOX2, OCT4, KLF4 and c-MYC, are sumoylated [87,115,116,117,118,119,120,121,122,123,124,125]. In mouse embryonic fibroblasts, inhibition of global sumoylation by UBC9 silencing hampers the efficiency of iPSC generation [126].

The murine DPPA2/DPPA4 dimer is a master transcription factor that controls early embryogenesis and zygotic genome activation. It also facilitates IPSC reprogramming, as well as transition of pluripotent mESCs towards a two-cell-like state resembling the totipotent two-cell stage [127,128,129]. DPPA2 sumoylation by PIAS4, a SUMO E3 ligase, negatively regulates its activity, inhibiting the two-cell-like transcriptional program. Phenotypically, overexpression of PIAS4 or that of a SUMO2∆GG-DPPA2 fusion (which is protected from SENP-mediated desumoylation) impairs early development of mouse embryos [130]. Recent findings revealed that PML NBs can enhance sumoylation of the KAP1 transcriptional repressive complex, as well as that of the DDPA2 transcription factor in mESCs [131]. PML NBs can thus orchestrate the activation or inhibition of key factors that silence transposable elements or control totipotency genes, by modulating their sumoylation.

SUMOs’ indispensable roles in maintaining nuclear and chromatin integrity, overseeing transcription and chromatin remodeling and modulating pluripotency and lineage commitment are supported by animal models in which total loss of sumoylation invariably impairs embryonic development. For example, UBC9 knockout mice display gross defects in blastocyst formation, which results in embryonic mortality [132]. Similarly, loss of the UBC9 function in fruit flies, worms and zebrafish impairs embryogenesis [133,134,135,136,137]. Studies in mice also revealed non-overlapping functions for different SUMO paralogs during embryonic development; while SUMO1 or SUMO3 knockout animals are viable, ablation of SUMO2 leads to embryonic mortality [138,139,140]. Interestingly, SUMO2 is the most abundantly expressed SUMO gene in early embryogenesis and its absence cannot be compensated by SUMO1 or SUMO3, supporting a developmentally essential role for this specific paralog. Meanwhile, many groups also strived to understand the contribution of other components of the SUMO pathway to development. Ablation of the SUMO protease SENP1 or SENP2 was found to be embryonically lethal in mice, with major effects on erythropoiesis and trophoblast development, respectively [141,142,143]. The PIAS family of E3 SUMO ligases contains several members that were initially identified as transcriptional co-regulators in STAT signaling, but today are known to facilitate SUMO conjugation of a wide range of cellular proteins [144,145]. Consistent with an essential role of sumoylation in embryonic development, PIAS1/PIASy double-knockout mice also exhibit embryonic lethality, arresting between days E10.5 and E12.5 [146,147].

Another important regulator, Lin28, has recently emerged as a key stemness factor and is capable of enhancing the efficiency of IPSC generation [148]. Lin28 is an RNA-binding protein, renowned for its inhibitory effect on the let-7 family of microRNAs [148]. The latter can silence SUMO expression by targeting the 3’ UTR regions of all three SUMO paralogs, SUMOs 1, 2 and 3 [87]. In this regard, Lin28 overexpression causes an upregulation of cellular sumoylation via let-7 inhibition, suggesting that SUMO peptides may function as crucial downstream mediators of Lin28, promoting self-renewal and maintenance of stem cells [87].

## 4. SUMO in Cancer and Therapy

The notion that SUMO levels are delicately balanced in somatic cells, while being capable of readily modulating and overseeing stress responses, stemness and differentiation has significant implications for cancer biology.

In retrospect, developments in the field of AML research in the late 1990s helped establish the earliest connections between sumoylation and cancer pathology [3]. Acute promyelocytic leukemia (APL) was clinically defined in the late 1950s as a rare AML subtype, characterized by the expression of PML/RARA, a fusion oncoprotein, which not only impedes granulocyte differentiation by interfering with the nuclear receptor signaling pathways but also disorganizes PML NBs, hence disrupting PML-regulated senescence or apoptosis pathways [149,150,151]. Today, APL is universally regarded as a prime model for precision medicine. APL’s success story is special in that the availability of its curative targeted therapy, which consists of arsenic trioxide, a traditional Chinese medicine, and all-trans retinoic acid (ATRA), preceded our full comprehension of disease pathogeny, as well as our perspective on the operational mechanisms of these drugs [152,153,154]. When trying to solve the enigmatic power of arsenic in inducing an APL cure, researchers discovered that the drug targeted PML/RARA, initiating its destructive proteolysis, which revealed PML as the first target of the RNF4 enzyme [52,53,152]. According to this model, arsenic drives PML/RARA sumoylation, in particular on Lys 160, thereby effectively promoting RNF4 recruitment, ultimately leading to proteasomal destruction of this oncoprotein. Concomitant with the PML/RARA loss, arsenic also favors PML NB biogenesis driven from the untranslocated PML allele by facilitating its oligomerization [155,156]. Arsenic-induced loss of the driver oncoprotein is thus accompanied by rapid restoration of NB-regulated functions, among which the activation of a PML/p53/senescence axis results in the eradication of APL blasts [155,157]. The arsenic/retinoic acid combination has now become the standard therapy for APL, accomplishing relapse-free cures in more than 97.5% of the patients, without additional chemotherapy in the frontline [158]. Finally, it is worth mentioning that SUMOs are also directly implicated in the APL pathogenesis, as PML/RARA sumoylation on Lys 160 appears to be important for cellular transformation in this cancer [159]. Current evidence suggests that loss of Lys 160 sumoylation impairs DAXX recruitment to PML/RARA, thereby interfering with its transcriptional repressor activity; however future work is needed to clarify the involvement of different SUMO isoforms in this process, both ex vivo and in vivo in APL mouse models.

The APL success story has paved the way for the development of similar combination therapies for the treatment of other cancers, and improved our understanding of how these therapies really work at the molecular level. Adult T-cell lymphoma (ATL) is an aggressive cancer of CD4+ T-cells, observed in some individuals infected with the human T-cell lymphotropic virus type 1 (HTLV-1) [160]. The latter expresses a potent viral oncoprotein, Tax, which initiates and maintains cellular transformation. A combination regimen that incorporates arsenic, interferon alpha (IFN) and an anti-viral drug achieves relapse-free cancer remissions in ATL patients with the cutaneous form of lymphoma by inducing the proteasomal destruction of Tax. Tax degradation results in a massive loss-of-viability in transformed T-cells, representing a perfect demonstration of oncogene addiction [160,161,162]. To elaborate, these drugs induce Tax poly-sumoylation, leading to RNF4-mediated ubiquitylation and proteasome recruitment [163]. In arsenic/IFN-treated ATL cells, Tax rapidly concentrates in PML NBs where a SUMO paralog switch occurs: baseline Tax conjugation by SUMO1 is replaced by SUMO2/3, likely leading on to subsequent interactions with RNF4. In this therapeutic system, arsenic/IFN-induced Tax degradation was shown to be dependent on the SUMO peptides, RNF4 and PML itself [163]. Recently, Bossis et al. elegantly demonstrated that in chemotherapy-resistant acute myeloid leukemia (AML), chemosensitivity can be restored by global inhibition of sumoylation [164]. In addition, in some non-promyelocytic AMLs, targeting cancer cell sumoylation seems to be an effective strategy to sensitize cells to retinoic acid-induced differentiation therapy [165].

Today, the connection between SUMO and carcinogenesis extends beyond the leukemia field. Indeed, many oncoproteins are subject to sumoylation, which can enhance protein stability or transformative capacity [3,166]. Likewise, the list of sumoylated tumor suppressors, which includes PML, is rapidly growing [3,166]. Most cancers maintain a small population of resident cancer stem cells (CSCs), the self-renewal and expansion of which not only promotes tumor growth during the initial stages of carcinogenesis but can also cause relapse following therapy, therefore, subverting therapeutic efforts [167]. Unsurprisingly, upregulated Lin28 levels along with low let-7 expression are markers for CSCs in most cancers and were linked to a poor prognosis [168,169]. These cancer cells display increased levels of sumoylation compared to their differentiated counterparts [3]. In support of a role for sumoylation in CSC maintenance, UBC9 or PIAS1 silencing can cause substantial depletion of the CSC population in breast and colorectal carcinomas [170]. The pluripotent stem cell-related transcription factor MYC can act as a potent oncoprotein, the constitutive activation of which promotes carcinogenesis in many tumors [122]. Recent findings indicate that MYC is highly sumoylated, which interferes with its phosphorylation. Sumoylation enhances MYC stability by promoting interactions with JNK1 (c-Jun N-terminal kinase 1), which, in turn, precludes proteasomal degradation-inducing phosphorylation of MYC by GSK3β (glycogen synthase kinase 3 beta) [171]. Thus, sumoylation likely facilitates MYC-driven carcinogenesis. Reciprocally, via positive feedback loops, MYC further upregulates cellular sumoylation in MYC-driven B-cell lymphomas by increasing the transcription of SUMO cascade enzymes [172]. Within this framework, it is possible to argue that MYC-induced sumoylation promotes CSC survival in B-cell lymphoma. In line with this hypothesis, researchers recently found that genetic or pharmacological inhibition of sumoylation diminishes the survival of MYC-positive B-cell lymphoma cells in culture, while also impairing disease maintenance in mouse xenograft models [172].

Most cancers have complex genetic mutational patterns where multiple growth-promoting proteins, for instance, MYC and β-catenin, cooperate to promote survival and proliferation. β-catenin is a transcription factor oncoprotein, which functions downstream of the pro-growth signals relayed by the Frizzled receptor [173]. The latter leads to the stabilization of β-catenin and transcription of its target genes, including MYC [173,174]. Interestingly, β-catenin is modified by SUMO, which can prevent its ubiquitylation and degradation [175]. MYC-induced sumoylation thus can favor β-catenin stabilization, leading to constitutive transcriptional activation of the downstream pro-growth genes even in the absence of a WNT-Frizzled pairing. With that being said, MYC multi-sumoylation also triggers its RNF4-mediated degradation, and inhibition of MYC sumoylation by PIAS1 downregulation was proposed to increase its transcriptional activity [121].

HIF1 is another case in point where sumoylation augments the stability and/or activity of a tumor promoter [176]. In most solid tumors, HIF1 activation confers resistance to hypoxia by orchestrating a metabolic shift toward glycolysis (Warburg effect) [69]. In parallel, HIF1 also promotes angiogenesis and contributes to metastatic capacity [69]. As we discussed above, HIF1 stability can be increased as a result of VHL sumoylation, which disables the ubiquitin E3 ligase function of this tumor suppressor on HIF1 [70].

While these examples demonstrate that sumoylation can promote tumorigenesis, in other circumstances. it may oppose oncogenesis by either stabilizing and/or activating tumor suppressors, or by attenuating oncoprotein function. For example, the transcriptional activity of androgen receptor (AR), which drives prostate cancer, is inhibited upon modification of this protein by SUMO1 near its N-terminus [177,178,179]. On the other hand, the tumor suppressor p53 is stabilized through sumoylation [63,66,67]. The potent tumor suppressor PTEN is also subject to sumoylation. PTEN antagonizes the function of PI3K [180,181], which is either mutationally activated or overexpressed in many cancers to relay PIP3/AKT-mediated growth signals [180,182]. SUMO1 modification of PTEN at Lys266 facilitates its association with the plasma membrane, where it can dephosphorylate and deactivate PIP3, in turn, suppressing cell growth [183]. In PC3 prostate cancer cells, SUMO1-modified PTEN suppresses anchorage-independent cell proliferation and also opposes tumor growth in vivo in a xenograft model [183]. Thus, therapeutic targeting of sumoylation may also be beneficial in prostate cancer. IRC117539 directly binds AR in the ligand-binding domain, inducing its sumoylation, followed by ubiquitination and degradation [184]. Consequently, IRC117539-induced AR depletion achieves selective and potent loss-of-viability in AR-positive prostate cancer cell lines, suggesting that pharmacologic manipulation of sumoylation may be a viable treatment strategy in solid tumors as well. Another tumor suppressor that is commonly mutated in cancers is BRCA1 [185]. BRCA1 participates in DNA double-strand break repairs where it functions as a ubiquitin E3 ligase [186]. Conjugation of BRCA1 by SUMO was found to upregulate its E3 ligase activity, in turn, expediting BRCA1-mediated responses to genotoxic stress at damage-induced DNA foci [187].

Overall, these studies paint a rather complex picture of SUMO’s context-dependent multifunctional roles in oncogenesis, implying that therapies aiming at manipulating sumoylation for the purpose of cancer treatment may benefit from precisely targeting this PTM on specific oncoproteins or tumor suppressors, rather than globally modulating cellular sumoylation levels. Nevertheless, data so far are also consistent with a model where increased cellular sumoylation favors CSCs\ maintenance. Indeed, most cancer cells upregulate the expression of SUMO cascade components that would enable proteome-wide conjugation [117,188,189,190,191,192,193,194]. For instance, UBC9 expression is upregulated in colorectal, breast, lung, liver and central nervous system cancers, as well as in melanomas and multiple myeloma [166]. Furthermore, in some specific cases, UBC9 overexpression correlates with higher sumoylation levels in tumors compared to differentiated cells, and also with a poor prognosis and therapy response [166,190,191,192,193]. Aside from UBC9, other SUMO pathway components, including the individual subunits of the heterodimeric SUMO E1 enzyme or PIAS proteins, are also commonly upregulated in many cancers [166,194].

Thus, strategies aimed at downregulating sumoylation may be beneficial in eradicating the CSC compartment, and synergize with chemo- or differentiation therapies to yield better clinical outcomes. This was exemplified with chemotherapy-resistant AML or retinoid-insensitive AML cases in which sumoylation inhibition ameliorates clinical responses [164,165]. A number of molecules have now become available to target the SUMO cascade components. For example, Ginkgolic acid, a phenolic compound extracted from the leaves of the Ginkgo biloba plant, inhibits the SUMO E1 enzyme, effectively reducing cellular sumoylation [195]. Remarkably, in lung cancer cells, this compound can impair epithelial-mesenchymal transition (EMT), invasion and migration capacity, and in both colon and human liver carcinoma cells, it can oppose invasiveness [196,197,198]. Unfortunately, the solubility of Ginkgolic acid as well as its pleiotropic effects—such as interferences with histone acetylation or biosynthesis of pro-inflammatory lipids—hamper its use in vivo and medical prospects [195]. To overcome this problem, highly selective sumoylation inhibitors have been designed, with reduced off-target effects. For example, ML-792 is a soluble, specific SAE1/SUMO E1 inhibitor, potent at nanomolar concentrations [199]. Treatment with ML-792 results in mitotic disruption and chromosome segregation defects, inducing substantial viability loss in colon, breast and melanoma cancer cell lines [199]. The therapeutic potential of ML-792 (hence, targeting sumoylation) is particularly remarkable in cancers bearing MYC mutations or amplifications. In accordance with the earlier findings that MYC-induced sumoylation is a therapeutic Achilles’ heel in B-cell lymphomas or that SAE1/UBA2 silencing causes synthetic lethality in MYC-amplified breast cancers, multiple MYC-expressing cancer cell lines are exceptionally hypersensitive to ML-792 [172,199,200]. Because the MYC oncoprotein is notoriously difficult to target, global inhibition of sumoylation with ML-792 (or with other similar drugs in the pipeline such as TAK-981) deserves attention as a potential therapy in MYC-driven cancers [194,201,202].

## 5. SUMO in Neurodegenerative Disorders

Dysregulated sumoylation is also tightly linked with the pathology of neurodegenerative disorders [203,204]. Many functionally and structurally distinct proteins are implicated in neurodegeneration, and accumulating evidence indicates that some of these proteins are exquisitely capable of undergoing liquid-liquid phase separation (LLPS) [205,206]. Phase separation of proteins facilitates the formation of molecular and subcellular assemblies, such as membrane-less organelles. These protein assemblies exhibit distinct material properties from their surrounding environment and have well-defined dynamics. Proteins that are subject to LLPS comprise some of the key players in the field of neurodegeneration, including Tau (implicated in Alzheimer’s disease), TDP43 and FUS (implicated in amyotrophic lateral sclerosis), Huntingtin (implicated in Huntington’s disease) and α-synuclein (implicated in Parkinson’s disease) [206,207]. Recent studies with these proteins revealed that disease-associated mutations or changes in their PTM profile cannot only facilitate LLPS but also enable a transition from a liquid-like state to a solid-like one that generates toxic fibrillar aggregates [207]. Remarkably, these aggregates almost invariably contain SUMO and ubiquitin [208]. Indeed, their pathogenic constituents, i.e., Tau, the amyloid precursor protein (APP), SOD1, ataxin-1 and many others, are all modified by SUMO [209,210,211,212,213,214,215]. There is growing evidence to suggest that sumoylation may be involved in the assembly, maintenance or disassembly of phase-separated proteins and membrane-less organelles, such as PML NBs, nuclear speckles, stress granules and the nucleolus [40].

There is an intricate relationship between neurodegeneration and SUMO in which the latter plays context-dependent roles depending on the proteins involved and the disease stage [3]. Sumoylation may favor LLPS by creating novel platforms for intramolecular SUMO/SIM interactions or hydrophilic interfaces for electrostatic attractions, thereby assisting the assembly of intrinsically disordered protein oligomers, eventually leading to aggregation [41]. In this case, SUMO functions as a ‘glue-like’ substance that enables the formation of cytotoxic aggregates, thereby playing a part in disease pathology.

For example, in Huntington’s disease, expansion of the N-terminal CAG repeats fabricates a long hydrophilic poly-Q stretch on the Huntingtin protein, which favors aggregation through intramolecular hydrogen bonding and electrostatic interactions. Huntingtin is subject to sumoylation by SUMO2/3, which is facilitated by PIAS1 [216]. This process further increases the aggregation tendency and toxicity of Huntingtin, likely by creating new electrostatic interfaces that favor molecular clumping [217]. In agreement with this observation, reducing PIAS1 expression protects from neurodegeneration in a Drosophila model of Huntington’s disease [216]. In other cases, sumoylation may stabilize aggregation-prone proteins by interfering with other PTMs, as we also discussed for oncoproteins or tumor suppressors. For instance, SUMO modification of Tau impairs its ubiquitylation, prevents degradation and facilitates the formation of toxic fibrillary tangles in neurons in Alzheimer’s disease [211,218]. Thus, in both cases (Huntingtin and Tau), sumoylation appears to have a detrimental effect by favoring neurodegeneration.

On the other hand, in specific cases, sumoylation may oppose pathogenesis by enhancing the solubility or turnover of aggregation-prone proteins. α-synuclein is a natively disordered protein, which is implicated in the pathology of Parkinson’s disease. Several mutations in α-synuclein, as well as increased gene dosage, were shown to increase its aggregation propensity and cause neurotoxicity [219]. Krumova et al. demonstrated that sumoylation dramatically increases α-synuclein solubility under the same conditions where the unmodified protein readily forms fibrils [220]. Furthermore, in a rat model of Parkinson’s disease in which α-synuclein is overexpressed in dopaminergic neurons, targeted ablation of its sumoylation sites exacerbates toxicity and neurodegeneration [220]. Ataxin-1 is another poly-Q protein, which is implicated in the pathology of spinocerebellar ataxia. Here, contrary to Tau, sumoylation of ataxin-1 promotes proteasomal degradation by facilitating its ubiquitylation, thereby reducing the aggregate burden [62].

While drugs altering CSC sumoylation may impair self-renewal and clinically synergize with conventional cancer therapies, pharmacologic induction of neural stem cell (NSC) expansion may be beneficial in neurodegenerative diseases. The generation of new neurons is a lifelong process. Although gliogenesis (generation of new glial cells) is common throughout adult life, adult neurogenesis is limited, with the persistence of a small number of NSCs in special regions of the brain [221]. Induction of NSC activity may facilitate the replacement of lost or degenerated neurons with new ones and result in functional recoveries in neurodegenerative diseases [222,223]. SUMO-enhancing drugs that are capable of passing the blood-brain barrier, such as interferon alpha, may achieve NSC induction [87]. In addition, next-generation NSC therapies that rely on the creation of IPSCs and subsequent neural induction may benefit from genetic or pharmacologic manipulation of sumoylation, which may enhance reprogramming or differentiation efficiency.

Finally, PML NBs’ druggable nature, which is outlined above for ATL therapy, may potentially be exploited in neurodegenerative diseases. PML NB induction may consume aggregation-prone misfolded proteins, which are almost universally associated with SUMO and PML [3,57,62]. In that regard, the IFN component of the ATL therapeutic regimen effectively induces the clearance of mutant ataxin-7 from the brains of spinocerebellar ataxia 7 (SCA7) mouse models, resulting in clinically relevant neurological improvements [224].

## 6. Concluding Remarks

In the previous sections, we tried to summarize the emerging knowledge about the functions of sumoylation, a developmentally essential process, in the pathology of cancer and neurodegeneration. Sumoylation appears as a remarkably versatile and stress-responsive PTM with context, timing and substrate-dependent roles, and it has the capacity to enforce distinct biochemical outcomes for specific proteins. Sumoylation targets some of the most important pathogenic proteins, and many diseased cells exhibit perturbed levels of sumoylation, which are normally finely balanced in healthy cells. Thus, sumoylation has attracted increasing attention from researchers in the fields of oncology, neurobiology and infection, as well as from clinicians. However, there is no simple linear relationship between sumoylation and pathology. Depending on which target protein(s) or signaling pathways are involved, sumoylation may play oncogenic or tumor-suppressive roles or have neuroprotective or neurodegenerative effects (Table 1). The pathogenesis of these diseases is often complex, meaning that both the consequences and relative contributions of sumoylation must be fully assessed to gain a better perspective on how SUMO-manipulating therapies might accomplish satisfactory clinical responses.

As pointed out above, the sumoylation system employs a limited set of enzymes. While this may be helpful in pharmacologically manipulating global cellular sumoylation in a proteome/chromatin-wide manner, targeted manipulation at the level of specific protein substrates will likely prove significantly challenging. Precise targeting of a given SUMO substrate may be achieved by a PROTAC (proteolysis targeting chimera)-like system (SUMOTAC) in which a small molecule can simultaneously bind and enforce proximity between UBC9, or a paralog specific SUMO E3 ligase or a SENP, and a target substrate whose sumoylation is to be modulated [225]. RNF4 plays essential roles in the stress response and DNA damage repair. It also mediates the clearance of a number of carcinogenic proteins, including MYC, Tax and PML/RARA, predominantly playing a tumor-suppressor role in these contexts. A potential SUMOTAC system may also engage RNF4 as a chimeric binding partner and exert significant therapeutic importance, as in APL. Multiple myeloma is a plasma cell cancer with a dismal prognosis, where resistance to dexamethasone has become a significant clinical problem. Du and colleagues recently proposed that sumoylation played a key role in dexamethasone resistance in multiple myeloma. Critically, inhibition of sumoylation by TAK-981 restored dexamethasone sensitivity in patient samples with relapsed disease, as well as in mouse xenograft models [226].

Although sumoylation is historically associated with nuclear proteins and processes, growing evidence suggests that this PTM also plays critical roles outside the cell nucleus. Sumoylation is emerging as a key regulator of autophagy, a cytoplasmic pro-survival mechanism that allows cells to clear damaged proteins and organelles. For example, sumoylation of PDPK1 (3-phosphoinositide-dependent protein kinase 1) negatively regulates autophagosome biogenesis and macroautophagy [227]. On the other hand, Beclin 1, which plays key roles in autophagy and cell death, is subjected to sumoylation by SUMO3 under starvation, which, in turn, enhances autophagy [228]. Deletion of SUMO1 in a Huntington’s disease mouse model or inhibition of sumoylation by Ginkgolic acid in human Huntington’s disease patient-derived fibroblasts strongly enhanced autophagy, promoting the clearance of toxic Huntingtin protein inclusions [229]. In *Caenorhabditis elegans*, the sole SUMO protein Smo-1 facilitates mitochondrial fission and mitophagy during aging. It promotes longevity by modifying critical regulators of mitochondrial homeostasis, among which is SKN-1, a homolog of mammalian Nrf2 that regulates redox metabolism [230]. These and several other studies prompt us to speculate that targeting sumoylation to modulate autophagy and mitophagy, possibly as part of combination regimens, might be therapeutically beneficial in neurodegeneration or cancer, and could even extend the lifespan. SUMO also modifies membrane proteins, ion channels and transporters [231], highlighting the emerging roles of this PTM in extranuclear processes. In this regard, N106 is a recently described small molecule SUMO E1 enzyme activator, which enhances sumoylation of the cardiac sarcoplasmic reticulum calcium ATPase SERCA2a, resulting in increased contractility in cultured cardiomyocytes and improved cardiac function in a mouse model of heart failure [232].

**Table 1:** Context- and substrate-dependent roles of sumoylation in cancer and neurodegeneration. Depending on the modified target protein, sumoylation may play a detrimental or beneficial role, both in the pathology and therapy of various diseases.

Disease or Physiologıcal Process.	Protein(s). Involved	Role of Sumoylation	References
Acute promyelocytic leukemia (APL)	PML/RARA	Arsenic-enhanced PML/RARA sumoylation promotes degradation and underlies the therapeutic effect of this drug in APL	[52,53,150,152]
cute promyelocytic leukemia (APL)	PML/RARA	K160, a major sumoylation site on PML/RARA is essential for cellular transformation in APL	[159]
Prostate cancer	Androgen receptor (AR)	Modification by SUMO1 attenuates AR’s transcriptional activity	[177,178,179]
Prostate cancer	Androgen receptor (AR)	IRC117539-induced AR sumoylation induces AR degradation and causes loss-of-viability in prostate cancer cell lines	[184]
Adult T-cell lymphoma (ATL)	Tax	The arsenic/interferon combination regimen induces Tax sumoylation, followed by degradation, causing loss-of-viability in transformed ATL cells	[163]
Various cancers	P53 and regulators	Collective sumoylation in situ in PML NBs may serve to accomplish full P53 activation	[57,63,64,65,66,67]
Breast and ovarian cancer	BRCA1	Sumoylation upregulates BRCA1’s E3 ligase activity	[187]
Metastasis, angiogenesis, various cancers	VHL and HIF1α	During hypoxia, sumoylation inactivates VHL, causing HIF1α stabilization	[70]
B cell lymphoma	MYC	Sumoylation enhances MYC stability; in MYC-driven B cell lymphomas, MYC further upregulates sumoylation; inhibition of sumoylation diminishes the survival of MYC-driven B cell lymphomas	[171,172]
Colon and various cancers	β-catenin	Sumoylation prevents β-catenin ubiquitylation and degradation	[175]
Cancer stem cells, various cancers	SUMO E1 and E2 enzymes, various SUMO E3 ligases (i.e. PIAS proteins)	Upregulated expression levels possibly maintain cancer stem cells	[117,188,189,190,191,192,193,194]
Neural stem cells	UBC9, global sumoylation, interferons	Enhanced sumoylation may promote neural stem cell maintenance and neuronal recovery in neurodegeneration	[87,222,223]
Spinocerebellar ataxia	Ataxin 1	Sumoylation promotes degradation, reducing aggregate burden	[62]
Parkinson’s disease	α-synuclein	Sumoylation enhances protein solubility and opposes toxicity	[219,220]
Huntington’s disease	Huntingtin	Sumoylation by PIAS-1 increases aggregation tendency and toxicity	[216]
Huntington’s disease, autophagy	Huntingtin	Inhibition of sumoylation enhances autophagy, promotes Huntingtin clearance	[229]
Alzheimer’s disease	Tau	Sumoylation prevents ubiquitylation and degradation, thereby promoting aggregation	[211,218]
Various neurodegenerative diseases	SUMO/SIM interactions	May promote liquid-liquid phase separation, cytotoxic protein aggregation	[40,205,206]

Future studies employing new and highly-effective SUMO purification techniques, coupled with ultra-sensitive differential proteomic analyses of diseased cells, should aid in the identification of novel druggable targets, for which several frontline therapies may already exist, paving the way for the development of highly potent combination regimens.
